# Influence of Superhydrophobic Coatings on Turbulence and Vortical Structures in a Submerged Impinging Jet

**DOI:** 10.3390/nano15181407

**Published:** 2025-09-12

**Authors:** Delfino Cornejo-Monroy, Betania Sánchez-Santamaria, David Luviano-Cruz, Manuel Alejandro Lira-Martínez, J. C. García, José Omar Dávalos

**Affiliations:** 1 Instituto de Ingeniería y Tecnología, Universidad Autónoma de Ciudad Juárez, Ciudad Juárez 32310, Chihuahua, Mexico; delfino.cornejo@uacj.mx (D.C.-M.); betania.sanchez@uacj.mx (B.S.-S.); david.luviano@uacj.mx (D.L.-C.); manuel.lira@uacj.mx (M.A.L.-M.); 2 Centro de Investigación en Ingeniería y Ciencias Aplicadas, Universidad Autónoma del Estado de Morelos, Cuernavaca 62210, Morelos, Mexico; jcgarcia@uaem.mx

**Keywords:** impingement jet, superhydrophobic coating, PIV, flow behavior, turbulence, vorticity

## Abstract

The impact of liquid jets on solid surfaces is a critical hydrodynamic mechanism in applications like cooling and cleaning. Surface properties, particularly superhydrophobicity, can significantly alter flow development throughout the impingement process. This work uses particle image velocimetry (PIV) to investigate a submerged water jet impinging on smooth and superhydrophobic surfaces. The jet, with a 4 mm diameter (D), was operated at a Reynolds number of 4500 and a nozzle-to-surface distance of 10D. Results demonstrate that the superhydrophobic surface (SHS) modifies the flow behavior significantly. Compared to the smooth surface, the peak jet velocity on the SHS increased by 26% in the axial direction and 19% in the radial direction. Furthermore, turbulent kinetic energy (TKE) at the impingement point was substantially higher on the coated surface. These findings are attributed to reduced wall friction on the superhydrophobic surface, which enhances momentum retention and alters turbulent production.

## 1. Introduction

A submerged impinging jet is a flow configuration where a liquid stream is directed onto a surface perpendicularly, creating a localized region of intense momentum exchange. This phenomenon is fundamental to numerous industrial applications requiring enhanced transport phenomena, such as the cooling of electric motors, thermal management, and material processing [1,2,3]. The hydrodynamic behavior of these jets is complex, influenced by parameters including flow conditions, geometric configurations, and, critically, the properties of the impingement surface. Previous research has explored these factors. For instance, numerical studies have focused on thermal effects, identifying the formation of vapor blankets and strong vorticity after impact [4]. Experimental work using Particle Image Velocimetry (PIV) has characterized the flow fields of turbulent jets, quantifying velocity decay and the influence of co-flow configurations [5]. Other studies have investigated how impingement distance and surface shape affect wave formation, splashing, and pressure distribution, revealing the intricate nature of the interaction of the jet with the solid boundary [6,7,8]. These studies consistently show that the region near the surface is characterized by complex flow structures, including thin boundary layers with significant momentum diffusion, which are exacerbated in a submerged environment [9,10,11,12,13].

Previous studies have shown that modifying the wall surface can strategically influence flow characteristics. Superhydrophobic surfaces (SHS), which are designed to minimize liquid adhesion, offer a promising route for flow control. The entrapped air layer (plastron) at the fluid-solid interface induces a slip condition, which has been shown to reduce drag, delay flow separation, and lower shear stress [14,15,16,17,18]. In the context of impinging jets, research has shown that SHS can enhance momentum retention prior to detachment and reduce drag forces [19,20]. Other studies have focused on the impact on heat transfer, noting a reduction in the Nusselt number for superhydrophobic surfaces [21].

While prior work has established the effects of SHS on heat transfer and overall momentum, a detailed quantification of how these surfaces alter the turbulent structure, vorticity, and Reynolds shear stresses in the impingement zone is still lacking. Understanding these mechanisms is critical, as they are directly linked to performance in applications such as cooling and surface cleaning. The novelty of this work lies in using PIV to provide this missing insight. By comparing flow velocity, turbulent kinetic energy (TKE), vorticity, swirl strength, and Reynolds shear stress (RSS) between smooth and superhydrophobic surfaces, this study reveals the fundamental mechanisms by which SHS alters turbulence generation and momentum transport.

## 2. Materials and Methods

### 2.1. Experimental Facility

**Figure 1:**
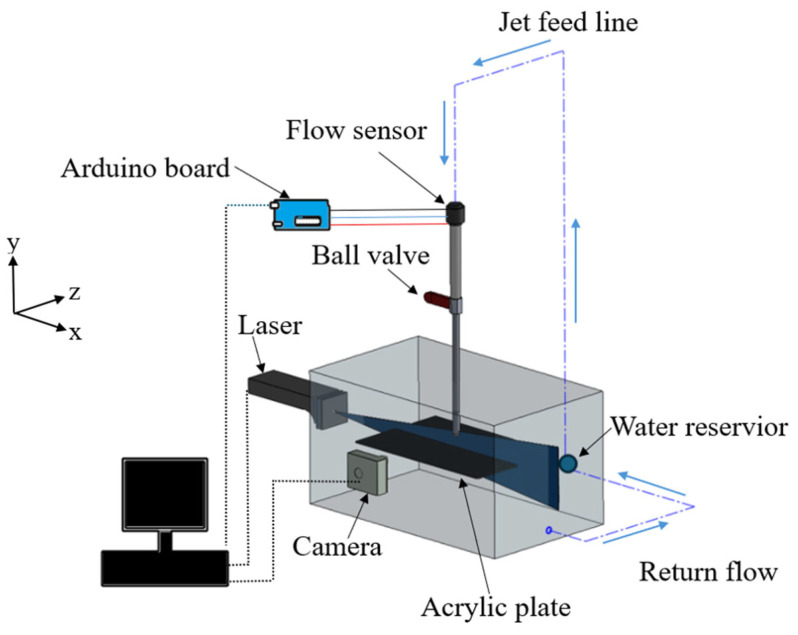
Schematic of the experimental facility. The setup includes a submerged impinging jet nozzle, a water tank with an acrylic test plate, a looped flow delivery system, and the Particle Image Velocimetry (PIV) system comprising a laser and a high-speed camera.

The experiments were conducted in a 50 × 30 × 27 cm water tank. The jet impinged perpendicularly on a 33 × 22 × 0.3 cm acrylic plate positioned at the midpoint of the tank and secured with suction cups. The flow was driven by a submersible pump (60 W, 58.33 L/min max flow rate) within a water reservoir connected to a looped piping system. The pump outlet was routed through a 1.27 cm diameter pipe, leading to a vertically oriented jet nozzle. See Figure 1.

### 2.2. Flow Control and Jet Configuration

The jet was issued from a circular brass nozzle with a length (L) of 46 cm and an inner diameter (D) of 0.4 cm. The resulting L/D ratio of 115 yielded a fully developed flow profile at the nozzle exit. The nozzle was positioned at a vertical distance of 10D above the acrylic plate. This distance avoids confinement at small distances and jet decay at large distances. A YF-S201 flow sensor (±2% accuracy, Sea, China), controlled by an Arduino board, was installed in-line to regulate the flow and to maintain a constant Reynolds number of 4500 for all experiments. This value ensures a fully turbulent jet at the nozzle exit and avoids the collapse of the plastron at higher Re.

### 2.3. Superhydrophobic Coatings Preparation and Characterization

**Figure 2:**
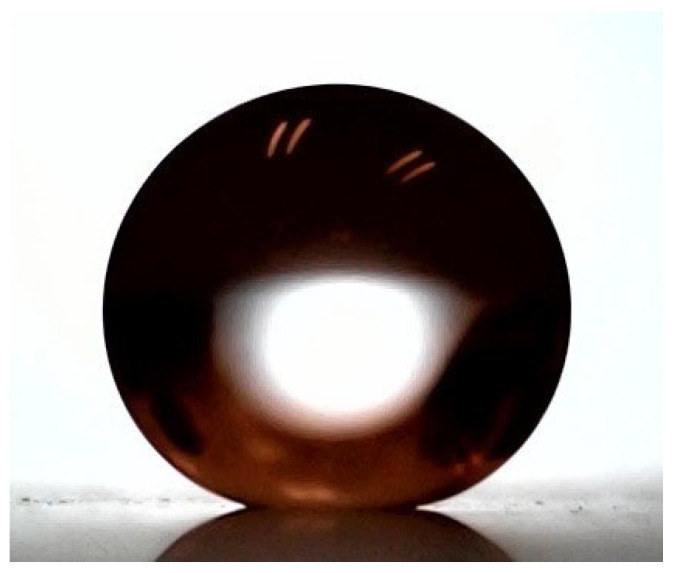
Wettability of the superhydrophobic (SHS) coating. A representative image of a 5 µL water droplet on the coated surface, which exhibited an average water contact angle (WCA) of 155.8° and a water sliding angle (WSA) of 3.1°.

The superhydrophobic coating was synthesized using a modified Stöber method [22]. The process involved preparing silicon dioxide (SiO_2_) nanoparticles (NPs) by adding the precursor tetraethyl orthosilicate (TEOS) to a solution containing isopropyl alcohol, deionized water, and an ammonium hydroxide catalyst. This mixture was stirred for 24 h for 40 °C. To impart hydrophobicity, the SiO_2_ NPs were then functionalized. This was achieved by adding hexane and 1H,1H,2H,2H-perfluorodecyltriethoxysilane (PFDTES) to the solution, followed by stirring for an additional 48 h at 60 °C [23]. The final superhydrophobic solution was applied to the acrylic substrates via a spray-coating technique at an air pressure of 30 psi at a distance of 15 cm. The resulting surface properties were characterized using custom-built equipment to measure the water contact angle (WCA) and water sliding angle (WSA) with 5 µL droplets of deionized water. The coated surface exhibited a WCA of 155.8° and a WSA of 3.1°, confirming its superhydrophobic nature. A representative image is shown in Figure 2.

#### Coating Durability Assessment

To evaluate the durability of the superhydrophobic coating, chemical stability tests were conducted based on ISO 2812-2 [24]. Coated glass substrates were immersed for 24 h in various aggressive solutions, including NaCl solutions (1, 3, 7, and 10%), solutions of varying pH (3, 7, and 11), and hydrogen peroxide solutions (3, 10, 20, and 30%). The water contact angle (WCA) and water sliding angle (WSA) were measured before and after exposure to assess the retention of superhydrophobicity.

The results, summarized in Table 1, show that the coating maintained excellent superhydrophobicity, with WCA values remaining above 154° across all conditions after 24 h of exposure. While the WSA increased slightly under the most aggressive conditions, such as 10% NaCl, the surface retained its hydrophobic functionality, demonstrating strong short-term chemical robustness.

### 2.4. PIV Measurement and Data Analysis

The flow field was characterized using a 2D PIV system. The water was seeded with 10 µm polyamide tracer particles. A blue diode laser (LD-PS/5, 450 nm, 5 W, Optolution, Lörrach, Germany) generated a light sheet aligned with the jet centerline, illuminating a 10 × 1 cm x-y plane. A high-speed Chronos 1.4 camera (Kron Technologies, Burnaby, BC, Canada) captured 500 image pairs for each case with an interframe time of 0.6 ms. A multi-pass FFT cross-correlation algorithm with window deformation was employed, using a final interrogation area of 28 × 28 pixels with 50% overlap, yielding a spatial resolution of 1.07 mm. The images were processed using the PIVlab 3.09 toolbox in MATLAB [25,26].

From the resulting velocity fields, the following flow characteristics were calculated: TKE, root-mean-square vorticity ($\omega_{rms}$), swirl strength ($\lambda_{ci}$), which identifies the strength of pure rotation in the flow and Reynolds shear stress ($\tau_{xy}$), defined, respectively, as:

(1) $$TKE=\frac{1}{2}\left(u^{\prime 2}+v^{\prime 2}\right),$$

(2) $$\omega_{rms}=\sqrt{\langle \omega^{2}\rangle },$$

(3) $$\lambda_{ci}=I(\lambda ),$$

(4) $$\tau_{xy}=-\rho \langle u^{\prime }v^{\prime }\rangle ,$$

Here, $u^{\prime }$ and $v^{\prime }$ are the fluctuating velocity components, ω is vorticity, λ represents the eigenvalues of the velocity gradient tensor, and ρ is the fluid density.

## 3. Results and Discussion

To describe the flow behavior, the results are presented in terms of ensemble-averaged contour plots and horizontal profiles. Each contour field corresponds to statistically converged data obtained from multiple independent PIV realizations, ensuring representativeness of the mean flow. Only half of the domain is shown due to symmetry. The horizontal profiles are extracted along four vertical positions: 0.25D, 0.5D, 0.75D, and 1D over the surface.

### 3.1. Velocity Field

**Figure 3:**
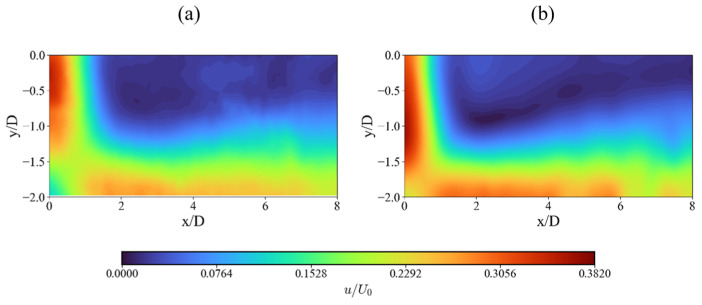
Comparison of non-dimensional averaged velocity contours: (**a**) SS and (**b**) SHS.

Figure 3 presents the non-dimensional contours of velocity. It is observed that at the impingement region, the SS case presents a reduction in velocity in both the axial and the radial directions. In the axial direction, the jet begins to decelerate approximately at 0.5D upstream of the impingement point for the SS case, while in the SHS case, the velocity decay starts closer downstream, at around 0.2D, corresponding to a 60% reduction in deceleration length. This deceleration suggests that momentum is transferred along the jet path. The SHS case increases the peak jet velocity by 26% along the jet centerline in comparison to the SS case. The reduced velocity decay in the SHS case suggests a diminution in wall friction promoted by the superhydrophobic coating. In radial direction, the jet accelerates along the plate, which is more pronounced in the SHS case. The non-dimensional velocity $u/U_{0}$ at the centerline reaches approximately 0.16 in the SS and 0.23 in the SHS. As the flow redirects radially, the SHS continues to promote higher velocities. The non-dimensional velocity ($u/U_{0}$) increases from the centerline to a maximum value of approximately 0.31 for the SHS case, compared to only 0.26 for the SS case. This sustained velocity gain confirms that the SHS not only affects the initial impact but also enhances momentum retention as the flow spreads across the surface. Such momentum preservation is significant because it represents a reduction in energy losses, a fundamental mechanism that is key to the efficiency of impinging jet systems.

**Figure 4:**
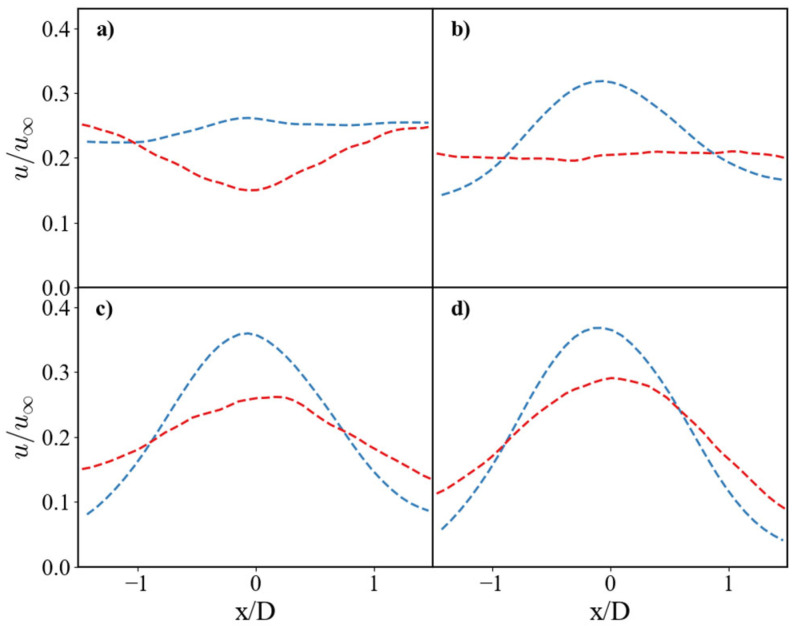
Mean Horizontal velocity profiles at different heights above the surface. The non-dimensional is compared between the SS (red dashed line) and the SHS (blue dashed line). Profiles are shown at vertical distances of (**a**) y/D = 0.25, (**b**) y/D = 0.5, (**c**) y/D = 0.75, and (**d**) y/D = 1.0.

The mean horizontal velocity profiles at different heights (Figure 4) further illustrate this. At distances of y/D = 1.0 and y/D = 0.75 (Figure 4d,c), the SHS profile maintains a higher peak velocity. Closer to the surface at y/D = 0.5 (Figure 4b), the SS profile flattens significantly, while the SHS profile remains parabolic, although with a reduced centerline velocity. This difference highlights the increased viscous diffusion in the SS case, which redistributes momentum more widely, leading to a broader but slower flow region.

### 3.2. Turbulence and Vorticity

**Figure 5:**
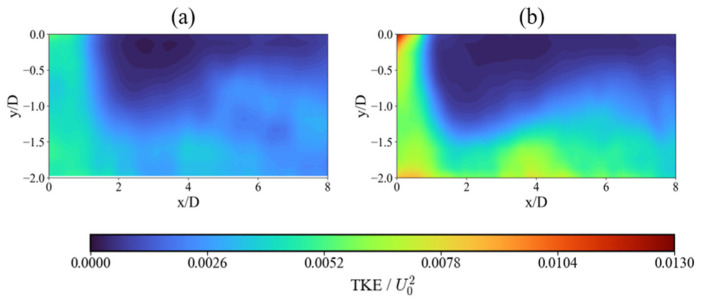
Comparison of non-dimensional averaged turbulence kinetic energy: (**a**) SS and (**b**) SHS.

The superhydrophobic surface strongly impacts the turbulent characteristics of the flow. Figure 5 shows that the mean TKE levels are dramatically elevated for the SHS case, approximately 90% higher in the jet core compared to the SS case. This increased turbulence persists along the jet path and extends radially. This phenomenon is twofold. First, the reduced friction on the SHS leads to less energy dissipation, preserving upstream turbulent fluctuations. Second, the slip velocity at the SHS boundary creates a large velocity gradient in the fluid layer immediately above the surface, which is a primary mechanism for turbulence production. At the microscale, this condition limits near-wall shear and suppresses energy dissipation, sustaining small-scale turbulent motions as the jet propagates downstream. In contrast, the no-slip condition on the SS dampens fluctuations near the wall, resulting in a less energetic turbulent field. Elevated TKE levels observed over the SHS suggest that reduced wall friction promotes more intense and widespread turbulent fluctuations. The intensified turbulence is relevant because enhanced mixing driven by turbulent fluctuations enlarges the active flow region, effects that are central to the performance of impinging jet systems in practice.

**Figure 6:**
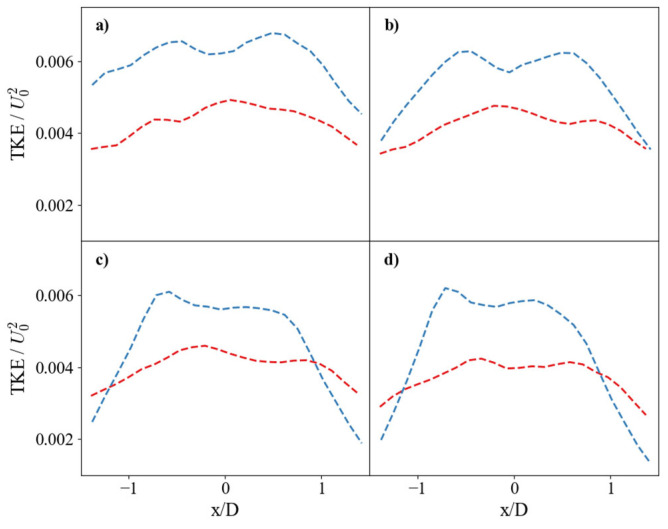
Horizontal profiles of turbulent kinetic energy (TKE). Comparison of non-dimensional TKE for the SS (red dashed line) and SHS (blue dashed line). Profiles are shown at vertical distances of (**a**) y/D = 0.25, (**b**) y/D = 0.5, (**c**) y/D = 0.75, and (**d**) y/D = 1.0.

As the flow approaches the wall (Figure 6a,b), TKE intensifies significantly more in the SHS case, increasing by 165% compared to just 18% for the SS case, confirming that the SHS actively enhances turbulence generation upon impact. This behavior suggests that turbulence becomes more intense and spreads outward as the jet interacts with the surface, with the superhydrophobic coating enhancing this effect by promoting stronger fluctuations near the wall and toward regions farther from the centerline.

**Figure 7:**
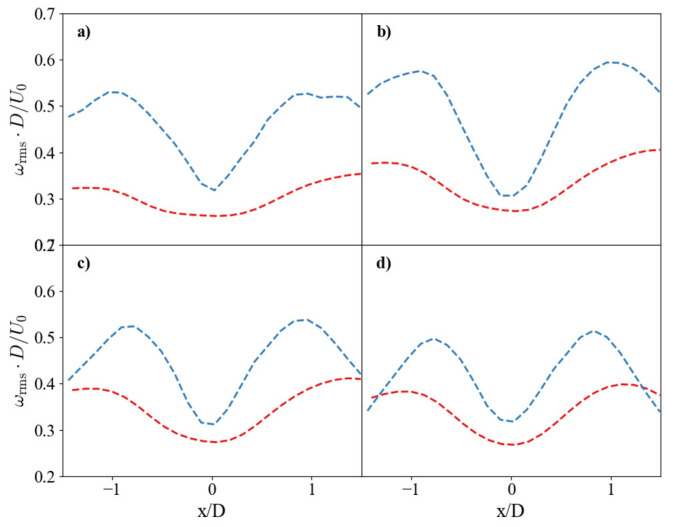
Horizontal profiles of root-mean-square (RMS) vorticity. Comparison of non-dimensional RMS vorticity for the SS (red dashed line) and SHS (blue dashed line). Profiles are shown at vertical distances of (**a**) y/D = 0.25, (**b**) y/D = 0.5, (**c**) y/D = 0.75, and (**d**) y/D = 1.0.

The root-mean-square (RMS) vorticity, shown in Figure 7, corroborates these findings. The SHS case maintains consistently higher RMS vorticity values, indicating stronger rotational fluctuations. For both surfaces, the fluctuation intensity is lowest at the impingement point and peaks symmetrically on either side of the jet axis. However, the peaks are located closer to the centerline for the SHS case (x/D ≈ ±0.8) than for the SS case (x/D ≈ ±1.2), suggesting a more compact and intense region of rotational activity.

**Figure 8:**
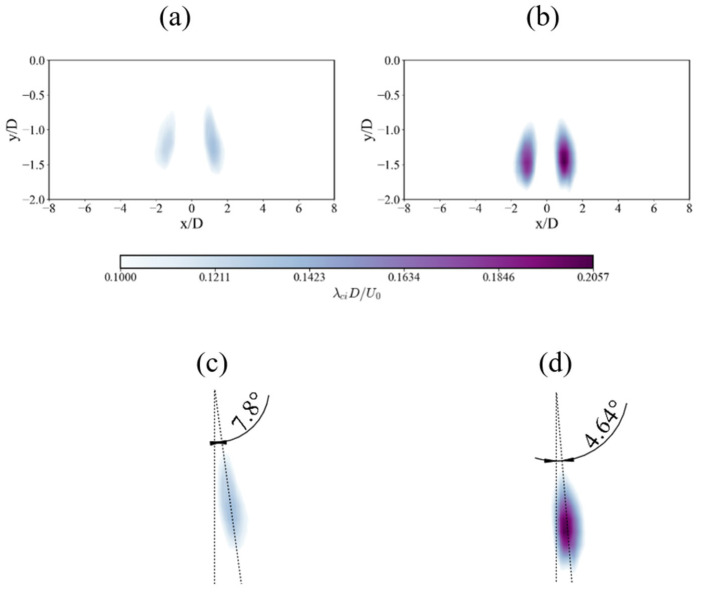
Comparison of non-dimensional averaged swirl strength contours: (**a**) SS and (**b**) SHS. The corresponding angular deviation of the dominant vortex cores is shown in (**c**) and (**d**), respectively.

The swirl strength contours in Figure 8 visualize the coherent vortical structures. Unlike the contour plots of other flow variables, which are presented over only half of the domain due to flow symmetry, both sides are displayed here to highlight the symmetric pair of vortices. These ensemble-averaged structures represent the dominant, stable vortex pair. The SHS case presents significantly higher swirl intensities, with a peak value nearly 70% higher than the SS case. Crucially, the spatial orientation of these vortex cores differs between the two surfaces. In the SS case, strong interaction with the wall causes the vortex cores to disperse radially, resulting in a wider angular deviation of 7.8° from the jet axis. In contrast, the slip condition on the SHS minimizes this wall interaction. This allows the vortex cores to remain more coherent and vertically aligned (4.64° deviation), preserving their energy and structure. The higher overall TKE on the SHS (Figure 5) reflects the sustained contribution of these more energetic and less dissipative vortical structures.

### 3.3. Reynolds Shear Stress

**Figure 9:**
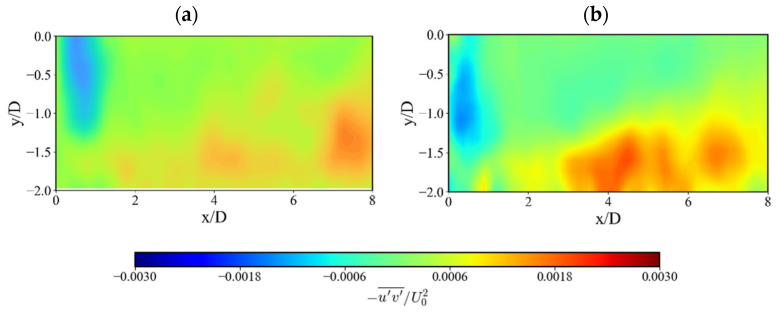
Comparison of non-dimensional averaged Reynolds shear stress contours: (**a**) SS and (**b**) SHS.

The mean Reynolds shear stress (RSS), which represents turbulent momentum transport, also shows distinct behavior for each surface (Figure 9). In the SHS case, there is a region of reduced shear stress immediately after impingement (from x/D = 0 to x/D = 2), followed by a region of much higher RSS further downstream. This suggests that while the SHS initially suppresses momentum exchange near the wall due to reduced friction, the preserved energy of the coherent vortices (as seen in Figure 8) promotes stronger turbulent mixing and momentum transport farther downstream. This observation indicates that the SHS not only alters the magnitude of RSS but also redistributes where the momentum exchange occurs.

**Figure 10:**
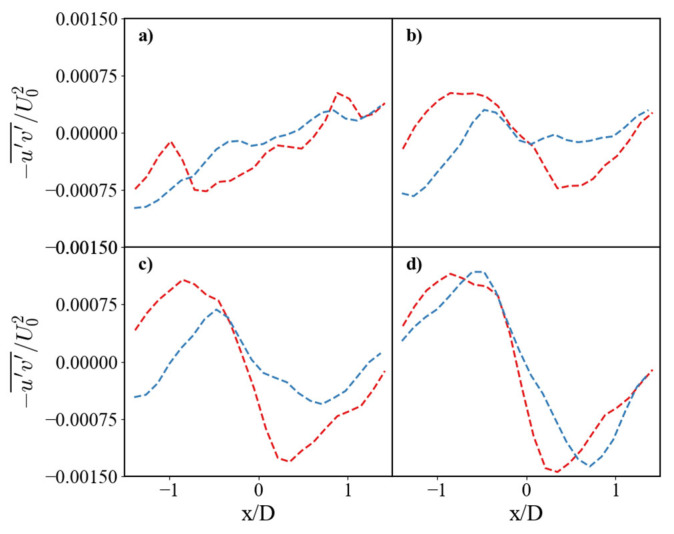
Horizontal profiles of Reynolds shear stress (RSS). Comparison of non-dimensional RSS for the SS (red dashed line) and SHS (blue dashed line). Profiles are shown at vertical distances of (**a**) y/D = 0.25, (**b**) y/D = 0.5, (**c**) y/D = 0.75, and (**d**) y/D = 1.0.

The mean RSS profiles in Figure 10 confirm this interpretation. Approaching the surface at y/D = 0.75 and y/D = 0.5 (Figure 10c,b), the RSS amplitude drops more significantly for the SHS case, indicating reduced momentum transport near the wall. This reduction is attributable to the lower wall shear enabled by the superhydrophobic coating. Conversely, the stronger RSS peaks observed for the SS case signify more intense turbulent momentum transport driven by higher surface friction.

## 4. Conclusions

This investigation experimentally analyzed the flow behavior of a submerged impingement jet on smooth and superhydrophobic surfaces. Using PIV, we compared velocity fields, TKE, vorticity, swirl strength, and Reynolds shear stresses.

The key findings are:

- The superhydrophobic surface significantly enhanced axial and radial velocities by reducing frictional losses at the wall.
- The SHS promoted substantially higher levels of turbulent kinetic energy, indicating that reduced surface friction leads to greater preservation of upstream turbulence and enhanced turbulence production at the wall.
- The SHS produced stronger vortical fluctuations and more coherent, energetic swirling structures that remained more compact and aligned with the jet axis.
- The SHS reduced Reynolds shear stress in the immediate impingement region but led to higher values farther downstream, indicating that the preserved energy of the flow structures promotes more intense turbulent mixing in the radial spreading region.

The most significant changes induced by the superhydrophobic surface were observed radially between x/D = −1 and x/D = 1 and axially up to y/D = 0.5 from the impingement point. These regions are critical for the development of flow structures over an SHS subjected to jet impingement. These insights into turbulence modification provide a fundamental basis that can guide the design of more efficient impinging jet systems with potential applications in energy, manufacturing, and industrial processes, among others. Future work should also include a thorough characterization of the mechanical properties of the coating, including standardized adhesion tests (e.g., ASTM D3359) and long-term durability under hydrodynamic stress, as well as its electrochemical properties, to fully assess its potential for industrial applications such as corrosion protection.

## Figures and Tables

**Table 1:** Water contact angle (WCA) and water sliding angle (WSA) of the superhydrophobic coating before and after 24 h immersion in various chemical solutions.

Measurement Stage	Property	NaCl Solution (%)				Solution pH			Hydrogen Peroxide (H_2_O_2_) Solution (%)			
		1	3	7	10	3	7	11	3	10	20	30
Initial State	WCA (°)	158.4	154.6	158.4	157.8	157.6	160.1	162.1	159.9	162.2	160.1	160.1
	WSA (°)	2.9	5.6	5.2	2.6	1.1	2.3	3.0	1.3	5.6	2.0	2.3
After 24 h	WCA (°)	156.1	154.6	154.6	154.1	159.1	155.2	155.3	157.8	157.0	156.9	156.5
	WSA (°)	5.8	7.5	6.9	18.2	5.5	8.5	12.5	2.1	9.6	4.1	5.7

## Data Availability

The original contributions presented in the study are included in the article; further inquiries can be directed to the authors.
